# Investigating the Two-Tiered System of Psychosocial Cancer Care in Brazil Using a Distress Screening Measure

**DOI:** 10.1200/JGO.2016.004978

**Published:** 2016-07-20

**Authors:** Cristiane Decat Bergerot, Errol J. Philip, Carolina Gaue Zayat, Isadora Miranda de Azevedo, Tereza Cristina Cavalcanti Ferreira de Araujo, Edvane Birelo Lopes De Domenico

**Affiliations:** **Cristiane Decat Bergerot**, **Carolina Gaue Zayat**, **Isadora Miranda de Azevedo**, and **Edvane Birelo Lopes De Domenico**, Universidade Federal de Sao Paulo, São Paulo, SP; **Tereza Cristina Cavalcanti Ferreira de Araujo**, Universidade de Brasília, Conselho Nacional de Desenvolvimento Científico e Tecnológico, Brasília, DF, Brazil; and **Errol J. Philip**, University of Notre Dame, Notre Dame, IN.

## INTRODUCTION

Brazil encompasses two epidemiologically distinct populations. The health care system follows this same paradigm, with public and private institutions coexisting side by side to serve two different populations. The Unified Health System was created in 1990 on the basis of the premise that health care is a fundamental right and that all Brazilian citizens and foreign visitors should have access to health care services.^1^ The private system preceded the Unified Health System and, despite now marketing itself as a complementary service to the public system, provides a host of overlapping services targeting those with greater socioeconomic resources.^2^

Almost one third of the population (those who have higher levels of education and who live in more developed areas) possesses health insurance.^3^ Studies have found a high prevalence of distress (36.4% to 65%) among Brazilian patients with cancer,^4,5^ regardless of care setting, a figure similar to that found in other developing countries^6^ and among patients with advanced cancer.^7^ However, in the public setting, the number of supportive care professionals is far outweighed by the number of patients requiring care, an issue that has led some institutions to implement systematic biopsychosocial screening to streamline services and provide care for those most in need.^4,8^

In addition to limited resources, cultural barriers to the development and implementation of effective and accessible psychosocial supportive care programs should be recognized. One such challenge may be the fact that many Brazilian patients perceive illness as possessing a spiritual component, both in its development and in its treatment. These beliefs can be a result of cultural traditions, as well as an erroneous understanding of disease processes and the potential for modern medical treatment.^9-11^ Furthermore, some individuals may become resigned to their diagnosis and engage in more passive or avoidant behavior, regardless of their prognosis. Past research highlights the prominent role of fear associated with cancer and the stigma associated with the misconception that cancer is untreatable.^12^ As elsewhere, adherence to cancer prevention guidelines, such as those that pertain to exercise and diet, as well as screening and follow-up, are far from optimal.^7^

This study sought to provide preliminary insight into the psychosocial care needs of patients in these two-tiered health care settings. A multicenter retrospective survey was conducted to determine whether (1) prevalence rates of psychosocial distress and symptom burden were similar and (2) whether patients reported different psychosocial or symptom-based needs on the basis of the institution in which they received care. It is hoped that this information will help elucidate gaps in psychosocial cancer care and inform supportive care practices.

## METHODS

### Sample

A total of 972 patients with cancer who were undergoing chemotherapy treatment were recruited at two oncology centers in Brazil: 580 patients from Centro de Câncer de Brasília, a private cancer center (PRI), which offers psychosocial, nutritional, and dentistry services, and 392 patients from Universidade Federal de São Paulo, a public university hospital (PUB), in which patients have access to psychological, psychiatric, social work, nutritional, and physiotherapy services.

### Procedure

The ethics committee of each site provided study approval. The study was conducted in compliance with the regulations of the ethical standards of the Helsinki Declaration and of Brazilian National Health Council Resolution No. 466/2012. Informed consent forms were signed by all participants. Eligibility criteria included being at least 18 years old, having been diagnosed with cancer, receiving chemotherapy treatment, being able to give written informed consent, and having an adequate level of functioning (Eastern Cooperative Oncology Group score ≥ 2).

Patients were recruited by a psychologist during their chemotherapy infusion and completed measures of distress, anxiety/depression, and quality of life (QoL). Data on sociodemographic variables, including patients’ age, sex, marital status, education, cancer diagnosis, and disease stage, were collected from patient records.

### Measures

#### Distress levels.

A self-report Brazilian Portuguese^13^ version of the National Comprehensive Cancer Network distress thermometer was used to assess distress level during the previous week on a visual analog scale, ranging from 0 (no distress) to 10 (extreme distress). This measure also identifies possible problems, grouped into practical, family, emotional, spiritual and physical problems, on a 35-item problem list.^13^ A cutoff score of 4 was used to indicate clinically significant distress.^13^

#### Anxiety/depression.

A Brazilian Portuguese version of the Hospital Anxiety and Depression Scale (HADS), a 14-item self-related scale, was used to assess symptoms of anxiety and depression. This scale is composed of two subscales, the HADS-A and the HADS-D, whose scores range from 0 to 21, with higher scores indicating greater levels of anxiety and depression.^14^ Scores ≥ 8 on the HADS-A and ≥ 9 on the HADS-D suggest clinical levels of anxiety and depression, respectively.^14^

#### Quality of life.

The Brazilian Portuguese version of the Functional Assessment of Chronic Illness Therapy-General, a 27-item self-related scale, was used to measure QoL across four domains of well-being (physical, social/family, emotional, and functional) on a 4-point Likert scale.^15^ The total Functional Assessment of Chronic Illness Therapy-General score is the sum of the scores for the four subscales. Scores range from 0 to 28 for the physical, social/family, and functional subscales, 0 to 24 for the emotional subscale, and 0 to 108 for the total score.^15^

### Data Analysis

Descriptive analyses were conducted to characterize patients from each institution. χ^2^ and *t* test analyses were used to determine potentially significant differences between groups across outcome measures. Statistical Package for the Social Sciences (SPSS) for Windows version 22 (SPSS, Chicago, IL) was used for all analyses.

## RESULTS

The majority (64.4% female; mean age, 55 years; 59.9% married) were diagnosed with GI (26.1%) and breast (25.6%) cancers, at an advanced disease stage (III to IV; 70.3%; Table 1). There were significant differences in demographic and illness-related variables between patients from both institutions. PRI patients tended to have more years of education (61.9% had a college degree) and were less likely to be diagnosed with advanced disease (57.5% *v* 71.7%).

**Table 1:**
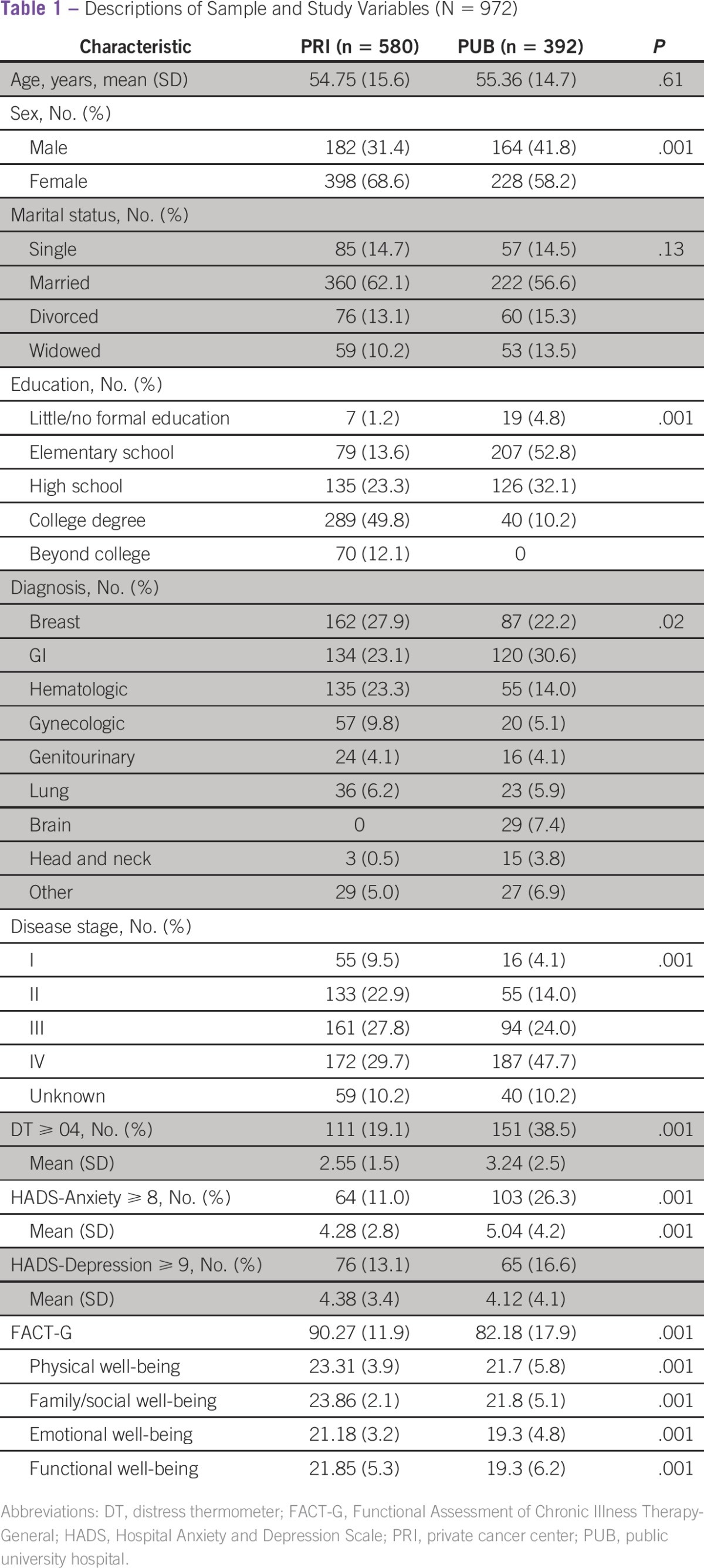
Descriptions of Sample and Study Variables (N = 972)

The prevalence of moderate to severe distress (distress thermometer ≥ 4) was significantly higher among PUB patients (Table 1). On the problem list (Appendix Table A1, online only), PUB patients tended to report significantly more practical problems (eg, financial, housing, and transportation). In contrast, PRI patients tended to report significantly more problems associated with family, emotional, and physical domains. For family problems, PRI patients more frequently reported dealing with children. In the emotional domain, depression, sadness, and loss of interest were more frequently reported by PRI patients, whereas fear, nervousness, and worry were more frequently reported by PUB patients. In the physical domain, the five most prevalent problems reported by PUB patients were fatigue, pain, dry/itchy skin, sleep, and nausea, whereas for PRI patients, the five most prevalent problems were appearance, sleep, fatigue, dry/itchy skin, and nausea. The prevalence of anxiety/depression was significantly lower for the PRI group, who also presented with a higher mean score on QoL compared with PUB patients (Table 1). This trend was reflected in higher scores on subscales; the US norm of physical well-being is at 50th (PRI) and 25th (PUB) percentile, social/family at 75th (PRI) and 50th (PUB) percentile, emotional at 50th (PRI) and 25th (PRI) percentile and functional at 75th (PRI) and 50th (PUB) percentile.

## DISCUSSION

This article explores the differences between two existing health care realities in Brazil and how these differences may affect the well-being of patients with cancer. There exists a paucity of psychosocial cancer research in developing countries and thus, the current study provides unique and important insight into the psychosocial needs of these different groups of patients. Our findings extend the current knowledge by documenting elevated levels of distress among patients with cancer in South America.

As hypothesized, individuals from poorer socioeconomic backgrounds (PUB patients) reported poorer QoL, more severe disease at diagnosis, and greater distress compared with those accessing care in PRI. Importantly, differences emerged in the types of psychosocial and physical problems reported by the patients, which may have implications for the structure of supportive care in these settings. However, the increased frequency of problem reporting was not reflected in subsequent impairments in QoL domains or higher rates of anxiety/depression. Furthermore, the comparison between the QoL scores and US norms highlights the need for developing interventions that target physical and emotional well-being at both institutions.

Because supportive care is provided free of charge to PUB patients, providers and resources are often overburdened. In line with international guidelines, biopsychosocial screening, shown previously to be feasible in Brazil,^4,5,13^ could assist in targeting limited resources to those in greatest need. Like many countries, Brazil is experiencing rising health care costs and increased demand for services.^16^ This has increased the need for local research endeavors that focus on the effective translation and implementation of international guidelines in care settings, with emphasis on cultural sensitivity, quality control, and cost effectiveness.

Of further interest is the relatively low frequency of spiritual problems reported by both groups. Given that the majority of Brazilians report a belief in God or a Supreme Being(s),^17^ one might expect that patients confronted by suffering and the possibility of death would seek comfort and support in their spiritual beliefs.^18^ As noted earlier, it may be that patients in both groups possessed a supportive spiritual community and thus did not report spiritual distress, or alternatively, they did not perceive the cancer care setting and associated professionals as appropriate sources of spiritual support. Whereas the discrepancies in QoL, distress, and disease stage are consistent with broader research examining socioeconomic status and health, no such data have been published in Brazil. In recognition of the paucity of research and clinical findings for psychosocial cancer care, this is an important step in describing patient needs and psychosocial outcomes. It is hoped that these data will increase public awareness of the importance of psychosocial care and the usefulness of validated tools to streamline services. Furthermore, it is hoped that these findings will provide an impetus for implementing quality care standards for comprehensive cancer care in Brazil and will help reduce discrepancies in the quality of psychosocial services and thus, patient outcomes.

The current findings must be considered in light of study limitations. Although this study did not set out to provide a comprehensive comparison of care and patient outcomes in the private and public cancer care settings, it does provide preliminary evidence of differences and may help guide future research efforts. It was not possible to collect detailed data regarding patients’ needs before their cancer diagnosis, whether these needs changed over time, or the impact of supportive care services accessed. This provides fertile ground for future research in the domain of psychosocial cancer care in developing countries such as Brazil.

In conclusion, this preliminary study suggests that patients diagnosed with cancer who access care in a public university hospital report impaired QoL, greater distress, and different supportive care needs than do those those in the private setting. It also highlights the potential influence of socioeconomic inequality on patient outcomes in the developing world and the challenges that exist in the promotion of well-being and health. In addition, this study further emphasizes the importance of providing comprehensive supportive care to all those affected by cancer, irrespective of socioeconomic status or care setting.

## Figures and Tables

**Table A1:**
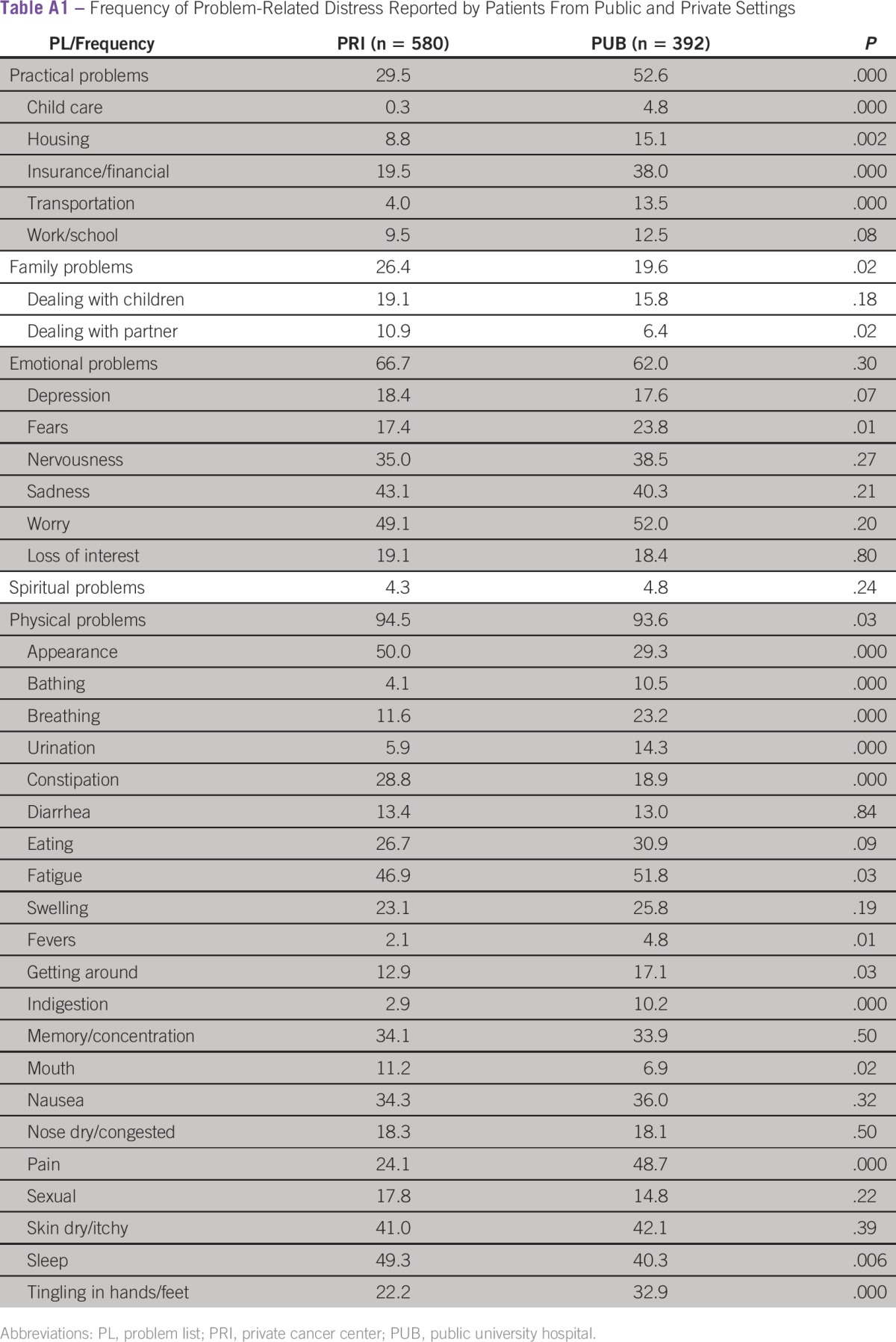
– Frequency of Problem-Related Distress Reported by Patients From Public and Private Settings

## References

[1] Ministério da Saúde. Portaria No 874/GM de 16 de maio de 2013. Diário Oficial da União. Ministério da Saúde, Brasil. http://bvsms.saude.gov.br/bvs/saudelegis/gm/2013/prt0874_16_05_2013.html

[2] Gerschman S (2013). Public and private health insurance in Brazil and European Union Countries. Am J Public Health Res.

[3] Instituto Brasileiro de Geografia e Estatística. Pesquisa Nacional de Saúde 2013: Acesso e utilização dos serviços de saúde, acidentes e violências: Brasil, grandes regiões e unidades da federação. Rio de Janeiro, Brazil, IBGE, 2015

[4] Decat CS, de Araujo TC, Stiles J (2011). Distress levels in patients undergoing chemotherapy in Brazil. Psychooncology.

[5] Lera AT, Miranda MC, Trevizan LLB (2011). Aplicação do instrumento termômetro de estresse em pacientes idosos com câncer: Estudo piloto [in Portuguese]. Rev Bras Clin Med.

[6] Ozalp E, Cankurtaran ES, Soygür H (2007). Screening for psychological distress in Turkish cancer patients. Psychooncology.

[7] Potash M, Breitbart W (2002). Affective disorders in advanced cancer. Hematol Oncol Clin North Am.

[8] Jacobsen PB, Holland JC, Steensma DP (2012). Caring for the whole patient: The science of psychosocial care. J Clin Oncol.

[9] Mello ML, Oliveira SS (2013). Health, religion and culture: A dialogue based on Afro-Brazilian customs. Saude Soc.

[10] Laplatine F (2001). Antropologia dos sistemas de representações da doença: Sobre algumas pesquisas desenvolvidas na França contemporânea reexaminadas à luz de uma experiência brasileira. In Jodelet D (ed). As Representações Sociais.

[11] Redko CP Religious idioms of distress in urban Brazil. Http://www.polbr.med.br/ano97/relig.php.

[12] Pinho AA, França I (2003). Prevenção do câncer de colo do útero: Um modelo teórico para analisar o acesso e a utilização do teste Papanicolaou [in Portuguese]. Rev Bras Saude Mater Infant.

[13] Decat CSA, Laros JA, Araujo TCCF (2009). Termômetro de distress: Validação de um instrumento breve para avaliação diagnóstica de pacientes oncológicos. Psico USF.

[14] Botega NJ, Bio MR, Zomignani MA (1995). Transtornos do humor em enfermaria de clínica médica e validação de escala de medida (HAD) de ansiedade e depressão [in Portuguese]. Rev Saude Publica.

[15] Arnold BJ, Eremenco E, Chang CH (2000). Development of a single Portugueses language version of the Functional Assessment of Cancer Therapy General (FACT-G) scale. Qual Life Res.

[16] Elzawawy A (2015). Could African and low- and middle-income countries contribute scientifically to global cancer care?. J Glob Oncol.

[17] Ipsos. Ipsos Global @dvisory: Supreme being(s), the afterlife and evolution. http://www.ipsos-na.com/news-polls/pressrelease.aspx?id=5217

[18] Wang GL, Hsu SH, Feng AC (2011). The HADS and the DT for screening psychosocial distress of cancer patient in Taiwan. Psycho-oncol.

